# Composition and toxicity of venom produced by araneophagous white-tailed spiders (Lamponidae: *Lampona* sp.﻿)

**DOI:** 10.1038/s41598-022-24694-5

**Published:** 2022-12-14

**Authors:** Ondřej Michálek, Andrew A. Walker, Ondrej Šedo, Zbyněk Zdráhal, Glenn F. King, Stano Pekár

**Affiliations:** 1 Department of Botany and Zoology, Faculty of Science, Masaryk University, Kotlářská 2, 611 37 Brno, Czech Republic; 2 Institute for Molecular Bioscience, The University of Queensland, St. Lucia, QLD 4072 Australia; 3 Australian Research Council Centre of Excellence for Innovations in Peptide and Protein Science, The University of Queensland, St. Lucia, QLD 4072 Australia; 4 Research Group Proteomics, Mendel Centre for Plant Genomics and Proteomics, Central European Institute of Technology, Masaryk University, Kamenice 5, 625 00 Brno, Czech Republic; 5 Faculty of Science, National Centre for Biomolecular Research, Masaryk University, Kamenice 5, 625 00 Brno, Czech Republic

**Keywords:** Proteomics, Transcriptomics, Ecology

## Abstract

Prey-specialised spiders are adapted to capture specific prey items, including dangerous prey. The venoms of specialists are often prey-specific and less complex than those of generalists, but their venom composition has not been studied in detail. Here, we investigated the venom of the prey-specialised white-tailed spiders (Lamponidae: *Lampona*), which utilise specialised morphological and behavioural adaptations to capture spider prey. We analysed the venom composition using proteo-transcriptomics and taxon-specific toxicity using venom bioassays. Our analysis identified 208 putative toxin sequences, comprising 103 peptides < 10 kDa and 105 proteins > 10 kDa. Most peptides belonged to one of two families characterised by scaffolds containing eight or ten cysteine residues. Toxin-like proteins showed similarity to galectins, leucine-rich repeat proteins, trypsins and neprilysins. The venom of *Lampona* was shown to be more potent against the preferred spider prey than against alternative cricket prey. In contrast, the venom of a related generalist was similarly potent against both prey types. These data provide insights into the molecular adaptations of venoms produced by prey-specialised spiders.

## Introduction

Venom is a trait used for various purposes, most notably predation and defence. It is a very successful trait, having evolved independently in a remarkably large proportion of animals on the tree of life^1^. Venoms represent complex mixtures of tens to thousands of compounds, including peptides, proteins and low molecular mass compounds^2,3^. While the complexity of venom composition can be driven by multiple factors^4^, diet is likely to have a strong effect on species that use venom for prey capture^5^. It has generally been assumed that predators with a narrower range of prey in their diets do not have to possess a complex venom arsenal. Indeed, it has been shown that specialised predators, like some spiders and cone snails, display a less complex repertoire of venom components compared to generalist predators^6,7^. Alternatively, predator–prey arms races are a very prominent factor determining the complexity of predatory venoms^8^ and may have a greater effect on specialists. As prey may evolve resistance to some venom components, predators may respond by recruiting additional toxins and thereby increasing venom complexity. In studies on arachnids, the venoms of specialist feeders are reported to be very potent on their focal prey, but less potent on alternative prey^9^. This suggests high prey-specificity of the compounds present in their venoms.

Spiders represent an ideal model group of predators for studying venom specificity. Although most spiders are generalist predators, approximately 5% of spiders are prey specialists^10^. However, spider-venomics research so far has focused predominantly on large species and those that are medically important, whereas many taxa have been neglected^11^. Although there is extensive literature on the diversity of disulfide-rich peptides found in araneomorph spider venoms, their venoms remain relatively unstudied compared with the vast diversity of araneomorph spiders, which represent ~ 90% of extant spider species.

Lamponidae is a relatively small spider family comprising less than 200 described species, mostly occurring in Australia and surrounding islands^12,13^. The most well-known are species of the genus *Lampona*, often called the white-tailed spiders. They are fairly large and often synanthropic. Due to frequent encounters, their capacity to bite through human skin, and their potent venom, these spiders have accrued an insidious reputation. They have been blamed for causing necrotic lesions, despite the complete lack of clinical evidence^14^.

In spite of their reputation, the venoms of Lamponidae have not been studied in detail. The pharmacological and enzymatic activities of venom from male and female *Lampona cylindrata* L. Koch, 1866 were shown to be different^15,16^, but the venom composition was not described comprehensively. *Lampona* spiders have been reported to prey on other spiders^12^. Our recent investigation of the prey-capture behaviour of *Lampona murina* L. Koch, 1873 revealed the presence of specific morphological and behavioural adaptations for handling spider prey^17^. Notably, *L. murina* relied on its venom to immobilise spider prey, suggesting that its venom may have arachnid-specific effects and may have been exposed to predator–prey arms races. Our previous study^6^ using proteomic profiling based on molecular mass revealed several dominant bands/peaks in *Lampona murina* venom, but the composition of the venom was not analysed in detail.

Here, our aim was to further investigate the venom composition and toxicity of *Lampona* spiders with a view to investigating the taxon-specificity of the venom. We hypothesised that *Lampona* venom is more potent on its focal spider prey, and that the spider-specific efficacy is due to specific venom toxins (either peptides or proteins). We employed proteo-transcriptomic methods to characterise the proteomic composition of venom produced by *Lampona* spiders, and compared its taxon specificity with that of a related generalist from the genus *Gnaphosa* (Gnaphosidae). We report that *Lampona* venom contains numerous and abundant peptides similar to those of other araneomorph spiders, as well as larger proteins. We confirmed that *Lampona* venom is more efficient toward spider prey than alternative prey.

## Results

### Proteo-transcriptomics

We collected *Lampona* spiders belonging to two species: *Lampona murina* and *Lampona* sp. indet. The venom of both species was pooled due to the low amount of milked venom, and subsequent venom activity was investigated at the genus level. Two separate transcriptomes were produced, corresponding to each species. Transcriptomic analysis recovered 152.3 million reads for the *L. murina* transcriptome and 165.3 million reads for the *Lampona* sp. transcriptome. The quality of the transcriptome was high as judged by a large portion of orthologs of the assembled transcripts mapping to two groups (Arthropoda: 60 species, 1066 orthologs; Metazoa: 65 species, 978 orthologs). The proportion of BUSCO orthologs identified by the assembled transcriptomes was 87% (Arthropoda) and 90.2% (Metazoa) for *L. murina*, and 83.9% (Arthropoda) and 88% (Metazoa) for *Lampona* sp. A high score of mapping of original reads to the assembled transcripts proved the high quality of the assembly (98.92% of aligned reads for *L. murina*, 99.35% of aligned reads for *Lampona* sp.).

**Figure 1:**
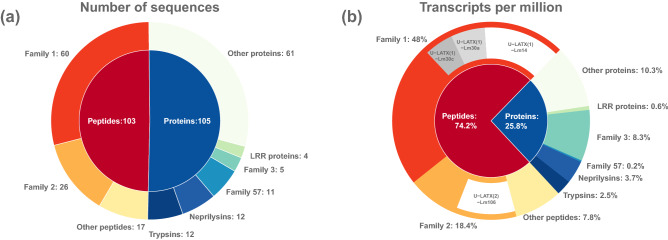
Diversity and abundance of components in the *Lampona* venom proteome. (**a**) The number of different components in the venom proteome. (**b**) Relative abundance of components in *Lampona* venom (based on TPM). The four most abundant toxins, each representing > 5% of total TPM, are highlighted.

To identify peptides and proteins in *Lampona* venom, we combined the transcriptome from dissected venom glands with LC–MS/MS proteomic analysis of crude milked venom. We decided to use only the *L. murina* transcriptome for this data analysis. All reported peptide and protein sequences were therefore confirmed to be present in the *L. murina* venom. The combined analysis revealed 208 different putative toxin sequences in the proteome after quality filtering (Supplementary Table S1). The venom contained numerous peptides with less than 100 amino-acid residues (103 sequences) as well as larger proteins (105 sequences) (Fig. 1a). The peptide-encoding transcripts were more abundant, accounting for 74.2% of the transcripts (Fig. 1b). Putative toxin families were assigned based on sequence similarities identified using BLAST and cysteine scaffold patterns in the case of disulfide-rich peptides.

### Peptides

**Figure 2:**
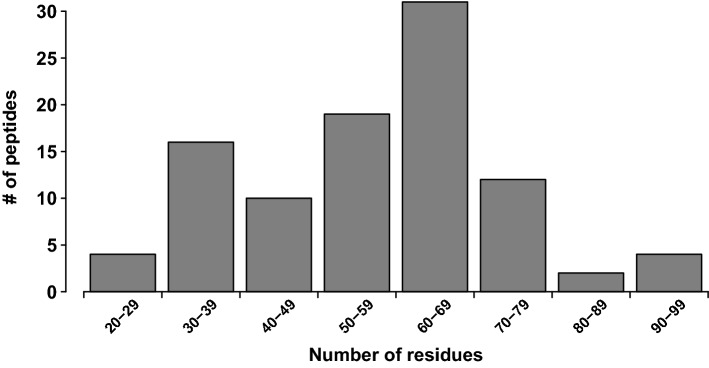
Size distribution of peptides identified in the *Lampona* venom proteome.

**Figure 3:**
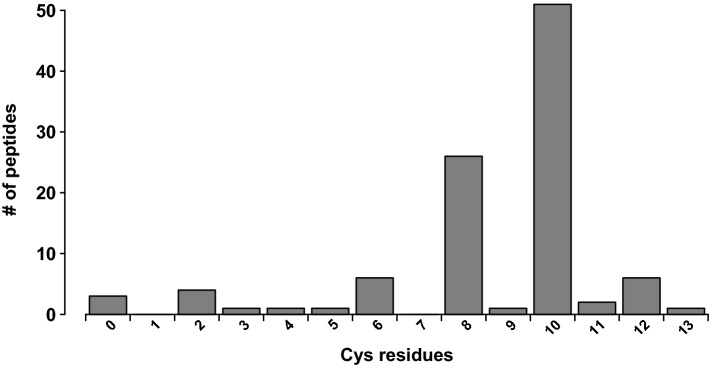
Distribution of peptides in the *Lampona* venom proteome according to the number of cysteines they contain. Note the high abundance of peptides with 8 or 10 cysteine residues.

The median length of the identified venom peptides (those with mature forms < 10 kDa) was 6.7 kDa (Fig. 2). Almost all identified peptides (97%) were cysteine-rich, with a cysteine scaffold suggesting they likely represent variations on the inhibitor-cystine-knot (ICK) motif. Interestingly, there were only two peptides with the basic spider-venom ICK scaffold (–C–C–CC–C–C–, where dashes indicating intervening non-Cys residues in variable numbers), as a large proportion of peptides contained more than 6 cysteine residues: 56% of venom peptides had 10 cysteine residues and 24% had 8 cysteine residues (Fig. 3). The identified peptides were classified into 15 families. Due to the complexity of previous spider toxin nomenclature, we did not attempt to extend previous classification systems and the 'families' depicted here are delineated only with respect to the sequence similarity of their members with other members of the same venom. The two most prominent peptide families were named lampotoxin venom Family 1 and Family 2.

### Lampotoxin venom Family 1

**Figure 4:**
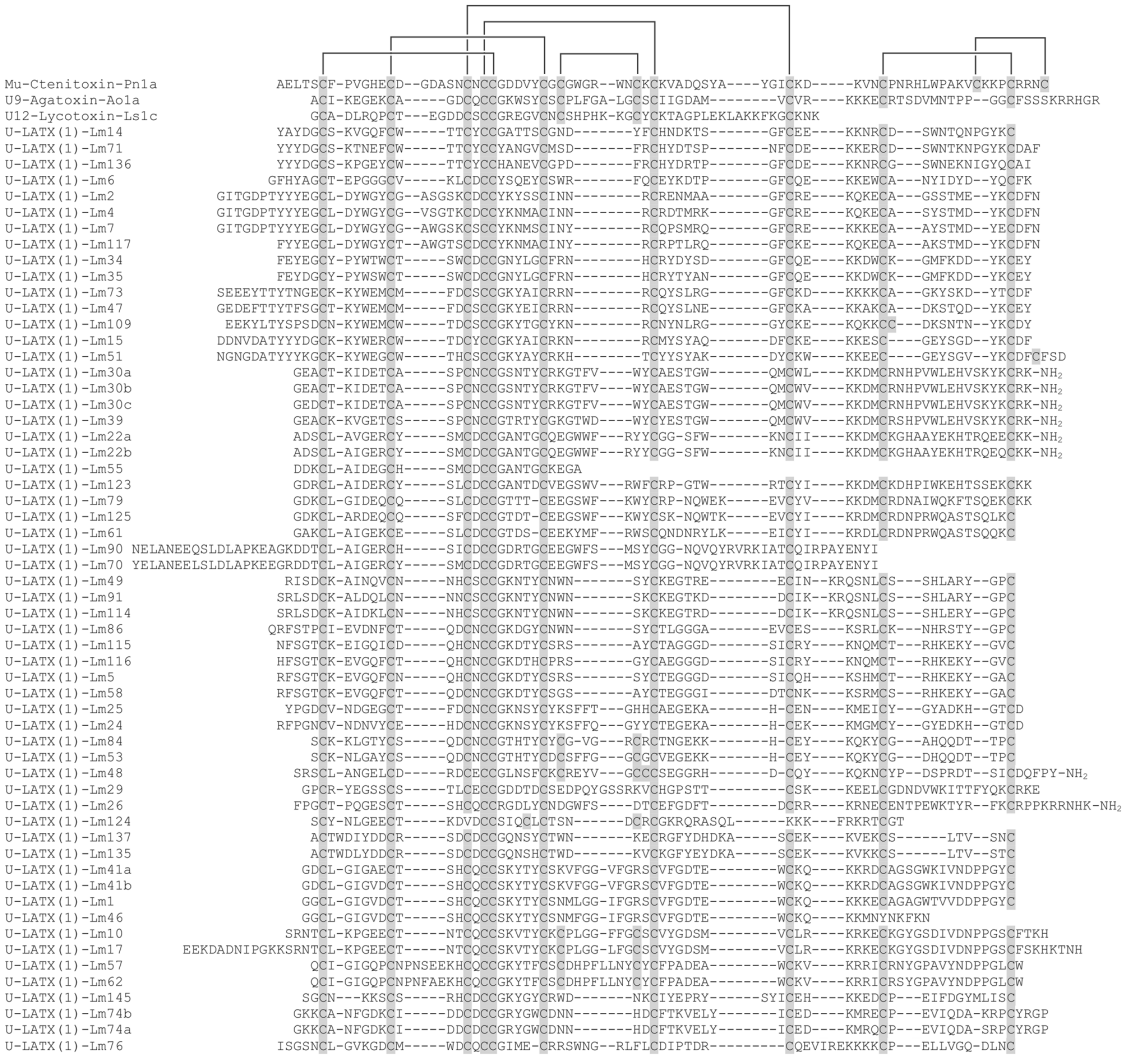
Sequence alignment of peptides in peptide lampotoxin Family 1 and the three closest BLAST matches. Cysteine residues are highlighted in grey.

Most toxins in Family 1 have ten cysteine residues with a scaffold of the form a –C–C–CXCC–C–C–C–C–C– (Fig. 4). Many Family 1 peptides are predicted to be members of Pfam Toxin_34 or D_CTX (δ-ctenitoxins) (Supplementary Table S1). Their cysteine scaffolds are similar to groups 6 and 8 of Na_V_-channel targeting peptides (NaSpTx) as classified by Klint et al.^18^, and to groups V–VIII of venom peptides of the ctenid *P. nigriventer* according to the classification of Diniz et al.^19^ (note that families in these two classification systems are not congruent). Similar described toxins from Ctenidae, Agelenidae, and Lycosidae are thought to be ICK peptides with long C-terminal extensions and extra disulfide bonds. While many of these known peptides have extra CXC motifs that yield a framework of –C–C–CXCC–CXC–CXC–C–C–C–, only seven identified lampotoxins contain these extra CXC motifs (Fig. 4).

We identified 60 different putative lampotoxins belonging to Family 1. Lampotoxin Family 1-encoding transcripts were the most abundant sequences in the transcriptome, representing 48.0% of all transcripts. Three of these lampotoxins (U-LATX(1)-Lm14, U-LATX(1)-Lm30a, U-LATX(1)-Lm30c) each accounted for more than 5% of all transcripts (Fig. 1b). Given the BLAST similarity of Family 1 toxins to µ-ctenitoxins, δ-ctenitoxins and ω-ctenitoxins, we hypothesise they may modulate the activity of voltage-gated sodium (Na_V_) or calcium (Ca_V_) channels.

### Lampotoxin venom Family 2

**Figure 5:**
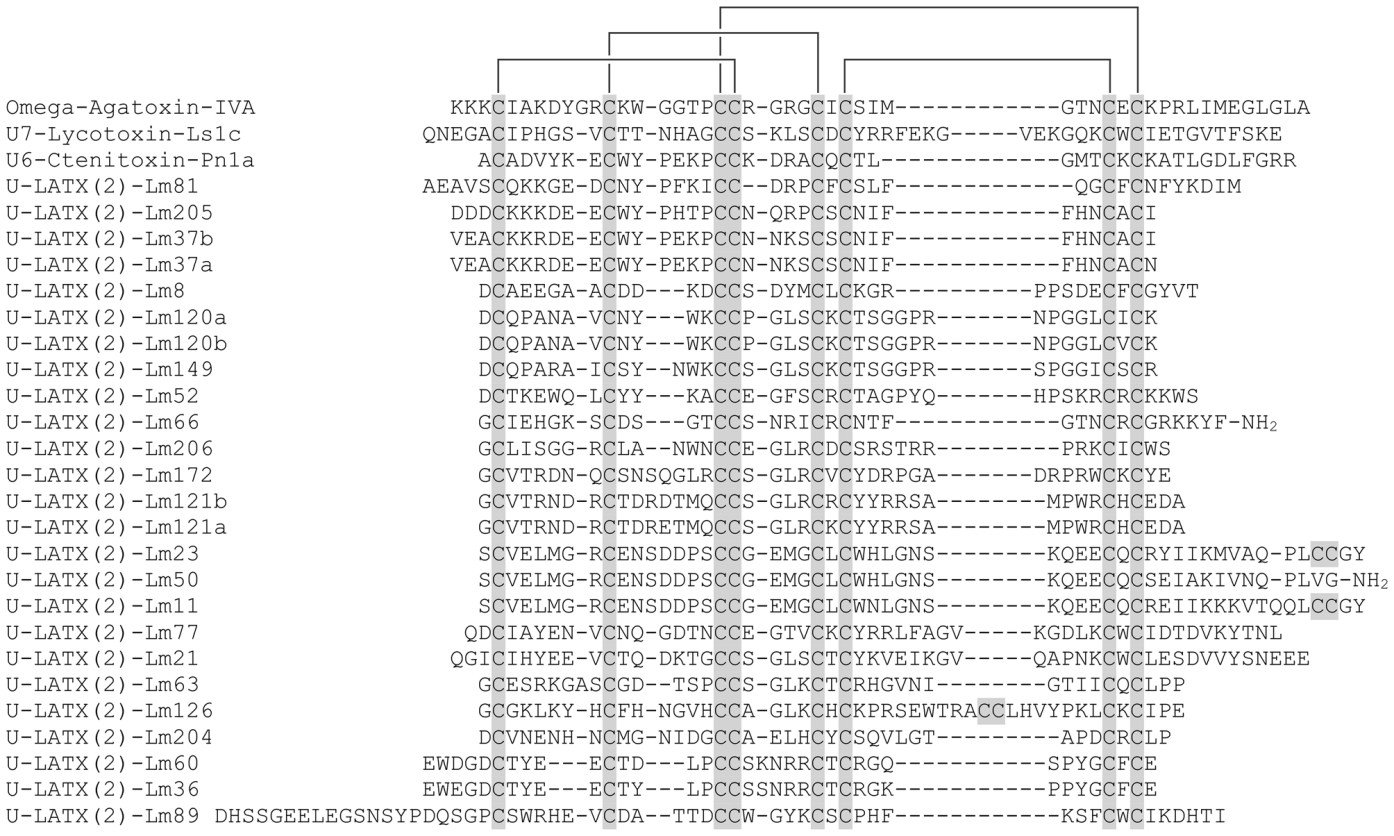
Sequence alignment of peptides in lampotoxin Family 2 and the three closest BLAST matches. Cysteine residues are highlighted in grey.

Most Family 2 toxins possess 8 cysteine residues in a scaffold lacking the CXCC motif of Family 1 peptides. However, they have two CXC motifs after the central CC doublet, without subsequent cysteine residues, yielding a scaffold of the form –C–C–CC–CXC–CXC– (Fig. 5). We identified 26 different putative Family 2 lampotoxins, which together contribute 18.4% of all transcripts. One lampotoxin (U-LATX(2)-Lm106) accounted for more than 5% of all transcripts (Fig. 1b). Family 2 lampotoxins show sequence similarities with ω-agatoxins, araneomorph venom peptides that modulate the activity of Ca_V_ channels.

### Proteins

The larger venom proteins were not as abundant as peptides, but they are very diverse, making up 57 different families (Fig. 1; Supplementary Table S1). The proteins comprised a number of putative toxins, including neprilysins, trypsins, LRR toxins, a phospholipase A_2_, and many others. In the terms of the number of sequences, the most numerous were toxins similar to trypsins from ctenid venoms with 12 sequences (Family 7). We also detected 12 protein sequences belonging to the neprilysin family (Family 9). The third most prominent protein family (Family 57) contained eleven protein sequences with little similarity to known toxins or proteins. Similarly, Family 3 contained five sequences with no detectable similarity to known proteins. Two additional sequences belonging to Family 3 were classified as peptides, as their molecular mass were lower than 10 kDa, and thus they were treated as such in the overall analysis (Fig. 1). Family 26 contained four different sequences similar to LRR toxins. In other protein families, only one or two sequences were identified in each family (Supplementary Table S1).

In terms of abundance, U-LATX(3)-Lm12, U-LATX(3)-Lm13 and U-LATX(4)-Lm28 were the most prominent proteins, forming 15%, 14% and 9% of protein transcripts, respectively. Proteins U-LATX(3)-Lm12 and U-LATX(3)-Lm13 belonged to Family 3, which is the most abundant protein family. The function of these proteins is not known, and they do not have close BLAST matches that could be used to infer their function.

Probably not all of 105 protein sequences in the *Lampona* venom are toxins. Some larger venom components are likely involved in toxin maturation (e.g. proline isomerase; U-LATX(33)-Lm184 and U-LATX(33)-Lm192; Family 33) or are venom spreading factors (e.g. hyaluronidase; U-LATX(30)-Lm108 and U-LATX(30)-Lm112; Family 30). A spermidine synthase was identified as well (U-LATX(19)-Lm157; Family 19), suggesting that the venom contains small-molecule polyamine toxins as do many other spider venoms^20^.

### The efficacy of crude venoms

**Figure 6:**
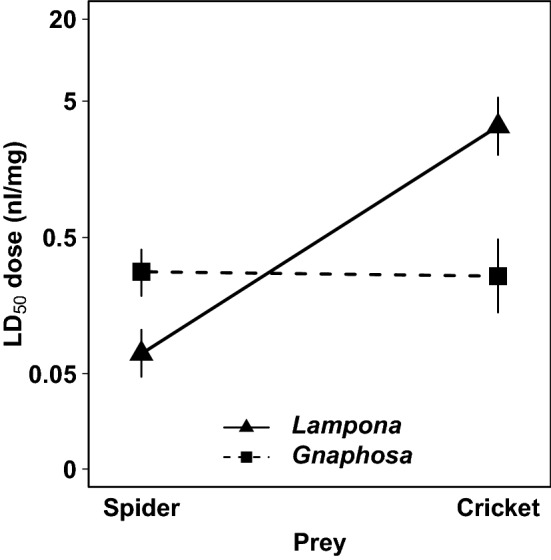
Comparison of median lethal doses (LD_50_) of crude venoms of *Lampona* and *Gnaphosa* spiders on two prey types measured 24 h after venom injection. Vertical lines represent 95% confidence intervals.

To test the relative efficacy of *Lampona* venom on different prey types, we injected venom into a preferred prey type (spiders) or non-preferred prey (crickets), and compared this with venom from a related generalist spider (Gnaphosidae: *Gnaphosa*). We observed lethal effects after 24 h and paralysing effects after 1 h (Table 1). The venom of prey-specialised *Lampona* was far more lethal on their preferred prey represented by a spider (LD_50_ = 0.07 nl/mg) than on alternative prey represented by a cricket (LD_50_ = 3.27 nl/mg). On the other hand, venom of the generalist predator *Gnaphosa* was similarly potent on both prey types (spider prey: LD_50_ = 0.28 nl/mg; cricket prey: LD_50_ = 0.26 nl/mg; Fig. 6 and Supplementary Fig. S1). The interaction between the predator species and prey type was significant (GLM-b, χ^2^_1_ = 43.8, *P* < 0.0001; Fig. 6), suggesting that the venom of *Lampona* sp. is prey-specific. The venom of *Lampona* was almost 50-fold more lethal to spiders than to crickets (GLM-b, χ^2^_1_ = 71.2, *P* < 0.0001), while the venom of *Gnaphosa* was similarly potent to both prey types (GLM-b, χ^2^_1_ = 0.1, *P* = 0.70, Table 1).

**Table 1:** Median lethal (LD_50_) and median effective (ED_50_) doses of crude venoms on two prey types.

	Preferred prey (spider)		Alternative prey (cricket)	
Spider species	LD_50_ (nl/mg)	ED_50_ (nl/mg)	LD_50_ (nl/mg)	ED_50_ (nl/mg)
*Lampona* sp.	0.07 (CI_95_ = 0.05, 0.10)	0.06 (CI_95_ = 0.04, 0.09)	3.27 (CI_95_ = 2.02, 5.31)	0.85 (CI_95_ = 0.59, 1.23)
*Gnaphosa* sp.	0.28 (CI_95_ = 0.19, 0.41)	0.05 (CI_95_ = 0.03, 0.07)	0.26 (CI_95_ = 0.14, 0.48)	0.05 (CI_95_ = 0.03, 0.08)

Numbers in brackets indicate 95% confidence intervals. Lethal doses were estimated from the mortality rates measured 24 h after injection; effective doses were calculated from the rates of affected prey 1 h after injection.

In *Lampona*, the paralysing dose after 1 h was similar to the lethal dose after 24 h for spider prey (GEE-b, χ^2^_1_ = 0.4, *P* = 0.51, Table 1); but it differed for cricket prey (GEE-b, χ^2^_1_ = 13.1, *P* < 0.001, Table 1): some crickets injected with lower venom concentrations were able to recover from paralysis after 24 h (Supplementary Fig. S2a). In *Gnaphosa*, the paralysing effect after 1 h was stronger than the lethal effect after 24 h for both spider and cricket prey (GEE-b, χ^2^_1_ = 52.5, *P* < 0.0001, Table 1), as lower venom concentrations paralysed prey, but they recovered after 24 h (Supplementary Fig. S2b).

### The efficacy of Lampona venom fractions

**Figure 7:**
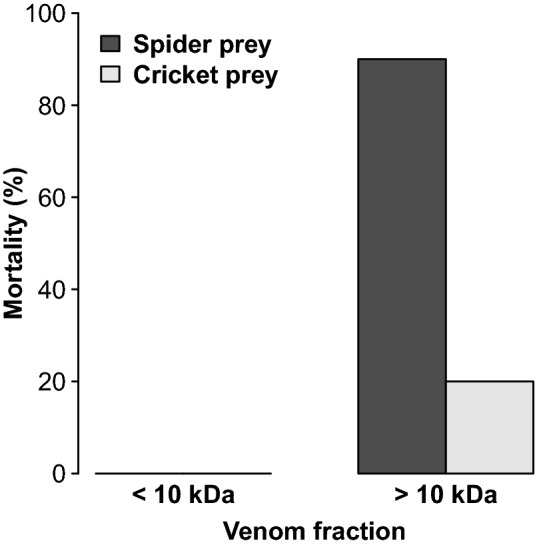
Comparison of the toxicity of venom fractions of *Lampona* spiders on two prey types measured 24 h after injection.

In addition, we tested the efficacy of low-MW and high-MW venom fractions. The toxicity differed between the high- and low-MW fractions (GLM-b, χ^2^_1_ = 19.8, *P* < 0.0001) and between prey types (GLM-b, χ^2^_1_ = 12.1, *P* < 0.001). The low-mass compounds (< 10 kDa) caused no mortality in either spiders or crickets, while the high-mass compounds (> 10 kDa) caused mortality but at different rates: 90% for spiders and 20% for crickets (Fig. 7). The effect of venom dose (after taking into account prey mass) was not significant (GLM-b, χ^2^_1_ = 0.9, *P* = 0.33). The mortality was similar after 1, 3 and 24 h for both combinations of fractions and prey.

## Discussion

In this study, we provided the first detailed analysis of the venom composition of prey-specialised white-tailed spider of the genus *Lampona*. Altogether, we confirmed the presence of 208 unique peptide and protein sequences in the pooled milked venom. The most numerous and abundant components of the *Lampona* proteome were peptides from lampotoxin families 1 and 2. The toxins from these two families share some similarities. Specifically, some toxins from Family 1 have the same two XCX motifs as Family 2 peptides, so the families are most likely related. Many of the described spider toxins from the infraorder Mygalomorphae contain six cysteine residues and form an ICK structural motif (but more complex toxins can be found in mygalomorph venoms as well^3^) in which two disulfide bridges and the intervening sections of the peptide backbone form a closed loop that is bisected by the third disulfide bridge. This motif provides these peptide toxins with exceptional resistance to heat, extremes of pH, and proteases^21^. Other structural scaffolds found in spider toxins include the disulfide-directed β-hairpin (DDH) and Kunitz motifs^22,23^. The lampotoxin Families 1 and 2 contained a higher number of cysteine residues, usually 10 or 8, respectively, and had a higher mass than most reported mygalomorph toxins. This was already implied by our previous study, where MALDI-TOF MS analysis of peptides showed peaks with the highest intensity close to 4 and 7 kDa^6^. It is possible that toxins with a more complex structure than the typical ICK motif, and containing more than six cysteine residues, are prominent in the Araneomorphae infraorder, although such a conclusion may be premature based on the current limited sampling of araneomorph spider venoms^24^. Indeed, these two lampotoxin families show complex cysteine scaffolds, similar to other toxins reported to exist in araneomorph venoms^24–29^. Cysteine-rich venom peptides are typically neurotoxins that target various ion channels^30^, suggesting a similar role for these *Lampona* peptides. Besides the Family 1 and 2 lampotoxins, most of the *Lampona* peptides (97%) also have a high cysteine content, suggesting the presence of an ICK motif or other disulfide-constrained scaffolds.

The *Lampona* venom proteins are more diverse than the venom peptides, although they are not the main venom component in terms of abundance. SDS-PAGE analysis of proteins performed in our previous study^6^ showed numerous dominant bands close to 37 kDa and between 50 and 75 kDa. Here, we found that *Lampona* venom contains proteins similar to galectins, LRR proteins, and trypsins (S1 proteases) that might correspond to the 37 kDa band, and toxins such as numerous neprilysins (M13 proteases) that might correspond to the 50–75 kDa band. The most numerous protein sequences in this study belonged to neprilysins (lampotoxin Family 9) and proteins similar to trypsins (lampotoxin Family 7). Neprilysins are proteins with functions connected to signalling pathways or metabolism of regulatory peptides^31^, but they have been also described as venom components of snakes and spiders^32–37^. Their proposed functions in spider venoms are extracellular matrix degradation^35,36^ or toxicity resulting in flaccid paralysis and darkened skin areas in cricket prey^37^. They may act as spreading factors in *Lampona* venom as well, but also facilitate toxicity since the high-mass compounds caused prey paralysis in venom fraction bioassays. The proteins classified as lampotoxin Family 7 showed similarity to trypsins or serine proteases. In most cases, their closest BLAST match was a serine protease from the spider *Phoneutria nigriventer* (Keyserling, 1891) (U21-ctenitoxin-Pn1a). Serine proteases in spider venoms have an unclear function, but it has been suggested that they play a role in toxin maturation, prey digestion, hemostasis impairment, or tissue damage^38,39^. We also identified four sequences similar to LRR proteins. LRR proteins have been described from a few spiders, namely the common house spider (*Parasteatoda tepidariorum* (C. L. Koch, 1841)), the western black widow spider (*Latrodectus hesperus* Chamberlin & Ivie, 1935), and the wasp spider (*Argiope bruennichi* Scopoli, 1772), but their role in the venom was not specified^40–42^. The most abundant components of the protein fraction were five proteins from Family 3 with unclear putative function due to low similarity to known sequences.

Behavioural experiments with *Lampona murina* showed that they rely on their venom to immobilise spider prey^9^. In our laboratory bioassays, the *Lampona* venom showed stronger potency toward spider prey, indicating the possible presence of prey-specific compounds in the venom. In our study, we revealed more than 200 venom components in *Lampona* venom. It is currently not known which compound or compounds are responsible for the prey-specificity of *Lampona* venom. Several reports of prey-specific venom compounds across different taxa can be found in the literature, including mollusc-specific toxins of a cone snail^43^, a bird-specific toxin of a mangrove catsnake^44^, and crustacean- and insect-specific latrotoxins in the venom of widow spiders (genus *Latrodectus*)^45^.

Very little information is known about the venom of spiders with specific dietary habits. The venom composition of fish-hunting spiders of the genus *Dolomedes* (family Pisauridae)^46^ and the ant spider *Lachesana tarabaevi* Zonstein and Ovtchinnikov, 1999 (family Zodariidae)^47^ have been investigated , but available information on the diets of these spiders suggest that they are not true prey-specialists (sensu Pekár and Toft^48^). Although many pisaurid spiders consume fish, they are generalists with broad diets^49^. Many zodariid spiders are specialists feeding only on ants, but some are generalists, including another species of *Lachesana*, *Lachesana insensibilis* Jocqué 1991^50^, and *Cybaeodamus taim* Lise, Ott & Rodrigues, 2009 with non-prey-specific venom^51^. Moreover, although the analysis of the venom composition of *L. tarabaevi* revealed interesting venom compounds with specific cytolytic activity^47^, there is no information on the prey specificity of these venom compounds.

The prey specificity of the toxins of prey-specialised predators has usually been indicated on a higher taxonomic level (e.g., phylum or class). Since *Lampona* venom was also potent against prey other than spiders, like crickets, although with lower efficacy, various compounds present in the venom may be effective against different prey taxa. Such contrasting taxon-specific potency of different toxins in a single venom has been already demonstrated in snake venom^52^. Alternatively, one toxin may exhibit different affinity and potency towards various prey taxa^53^. In our experiment, we tested the venom specificity only on one representative of the focal and alternative prey taxa due to the low amount of milked venom. The prey species utilised in this study are not part of the natural trophic niche in which *Lampona* venom has evolved. The prey species were selected due to their availability in high numbers. Additional experiments on the natural prey of *Lampona* spiders are required to further confirm the prey-specific venomic adaptations. The venom specificity of araneophagous spiders is usually not restricted to a single prey species, as they are not monophagous but indiscriminately hunt several species of spider prey^51^. Anecdotal observations of the white-tailed spiders suggest they are able to subdue and paralyse various spider prey taxa^12^. Our previous study^9^ has shown the paralysis latency of *L. murina* spiders is also longer in another cricket species (*Acheta domestica* (Linnaeus 1758)), confirming the pattern of lower venom susceptibility of alternative insect prey.

One limitation of this study is that we pooled the venom from two *Lampona* species due to the low volumes available, and analysed the venom only at the genus level. We analysed only the *L. murina* transcriptome against proteomic data obtained from the pooled venom sample and present it as a venom proteome of *Lampona* (species not defined). We note that it is technically possible that proteomic data from the unidentified *Lampona* species may have been used to 'misidentify' a sequence which is present in the *L. murina* transcriptome but not present in *L. murina* venom. However, considering the two species are congeneric they should share a large portion of almost identical transcripts, and we consider that the frequency of such false positives is likely to be very low in comparison to true positives. All *Lampona* spiders are generally considered to be araneophagous, therefore we expect similar venomic adaptations in the whole genus. Further investigation is needed to identify species differences in venom composition, spider-specific toxins and their mode of action.

Interestingly, the high-MW fraction from *Lampona* venom showed higher efficacy than the low-MW fraction in potency bioassays, although the peptides were more abundant than proteins in the venom as revealed by proteo-transcriptomic analysis. The discrepancy between the abundance of high-mass compounds and their efficacy might be caused by the methodology used for the venom fractionation. Specifically, due to the large size of the disulfide-rich peptides detected in *Lampona* venom (mean 6.7 kDa and many 9–10 kDa), and depending on the steepness and precision of the molecular weight cut-off used to separate high- and low-molecular-mass toxins, some disulfide-rich peptides may also contribute to the observed toxicity toward preferred prey, as they might not have been separated properly from the protein fraction used in the bioassays. More experiments are needed to identify the exact toxins responsible for the prey-specific efficacy of the venom, starting with a greater quantity of venom and employing chromatographic separation techniques. Some recent studies revealed that invertebrate venoms may exhibit inter-individual variability in venom composition^54^. Ideally, the venom from different individuals should be analysed separately. Here, we were not able to analyse *Lampona* venom on an individual level due to the low number of individuals and the small volume of venom obtained from each individual. Thus, we had to combine venom from several individuals for the analyses described herein. Although this is not ideal, our study still provides the first detailed insight into the venom composition of *Lampona* spiders.

Overall, our study revealed that besides morphological and behavioural adaptations, the white-tailed spiders also possess potent prey-specific venom to handle their focal spider prey. We identified a unique mix of novel compounds in *Lampona* venom. Due to its taxon-specific toxicity, *Lampona* venom represents an excellent model system for future studies focusing more thoroughly on the mechanisms of venom prey-specificity or possible exploitation of such toxins in applied research.

## Methods

### Spiders and prey

*Lampona* spp. spiders (Lamponidae) were collected on the Macquarie University campus, North Ryde, Sydney, Australia. Collected spiders belonged to two species (*Lampona murina* and *Lampona* sp. indet.); specimens of both species were used to analyse the venom on the genus level due to the low numbers of specimens and the small amount of milked venom. *Gnaphosa lucifuga* (Walckenaer, 1802) spiders (Gnaphosidae), were collected in Mohelno Serpentine Steppe in the Czech Republic. Spiders were kept singly in glass tubes (height: 6 cm, diameter: 1.5 cm) with moisturised gypsum on the bottom and stored in a chamber at room temperature (22 °C) and L:D = 16:8. Spiders were fed regularly with other spiders or crickets. Water was provided every three days.

In laboratory bioassays, we used juvenile *Pardosa* sp. wolf spiders (*Pardosa lugubris* group, N = 130, body mass: 13.0 ± 3.5 mg) and *Gryllus assimilis* (Fabricius, 1775) juvenile crickets (N = 125, body mass: 5.6 ± 4.0 mg) as prey. *Pardosa* spiders were collected in the surrounds of the Department of Botany and Zoology at Masaryk University (Brno, Czech Republic) and kept singly in punctured Eppendorf tubes placed in a bag with moisturized cotton in a chamber at low temperature (10 °C) and L:D = 16:8. Crickets were bought at a local pet store.

### Venom extraction

Venom was obtained from six *Lampona* spiders and five *Gnaphosa* spiders using an electrical milking technique^55–57^. A spider was anesthetised with CO_2_ for 2 min, placed on a stub, covered with a mesh and the venom collected into a glass microcapillary (volume 0.5 or 1 µl) that was slid onto one of the fangs of the spider’s chelicerae. The spider was stimulated with an electric impulse to release venom into the capillary. Individual spiders were milked several times at approximately three-weekly intervals. Microcapillaries containing the venom were stored in the freezer at − 80 °C before further processing. Overall, 5 μl of *Lampona* venom and 6.5 μl of *Gnaphosa* venom was obtained by repeatedly milking the spiders.

### Transcriptomics

To prepare a transcriptome library, paired venom glands were dissected from *Lampona murina* and *Lampona* sp. spiders that were milked five days before dissection to stimulate venom transcript production. To minimize contamination, the dissected glands were immediately placed in a drop of physiological solution (0.9% NaCl) and left for 5 min to wash away haemolymph and epithelial cells and other tissues on the surface of the glands. Then, glands were placed into 2 ml of TRIzol Reagent (Life Technologies, USA) in an Eppendorf tube, compressed by a tweezer and kept for 15 min at room temperature. The tube was stored at − 20 °C until further processing. Total RNA was extracted using TriReagent (MRC Holland) and RNAeasy spin columns (Qiagen). The tissue was lysed in TriReagent, and RNA was separated into an aqueous phase with 1–bromo–3–chloropropane (BCP) (MRC Holland). The aqueous phase was mixed 1:1 with 70% ethanol and transferred to an RNAeasy column. The extraction continued according to manufacturer’s protocol, including on-column DNAse digestion.

The libraries were sequenced on the Illumina Hi-Seq platform. Raw reads were quality checked (using FastQC^58^) and pre-processed before assembling (trimming by Trimmomatic^59^ and correction of sequencing errors by Rcorrector^60^). The following steps were repeated for both species (*Lampona murina* and *Lampona* sp.) to obtain two separate venom gland transcriptomes. Transcriptome assembly was performed using Trinity^61^ in three runs with different kmer setting (21, 25, 31) to optimise the results as advised by the Trinity authors. All standalone assemblies were combined and clustered based on nucleotide/amino-acid sequence similarity using the EvidentialGene toolset^62^. The quality of the reduced set of transcripts was assessed by mapping of pre-processed reads back to the transcripts using BOWTIE^63^ and by comparing them to specific ortholog databases (i.e., Arthropoda, Metazoa) using BUSCO^64^. Open reading frames larger than 90 bp were detected and translated by TransDecoder^65^ to yield a database of possible amino acid sequences to compare to mass spectrometric data.

### Proteomics

Crude pooled *Lampona* venom (2.25 µl) was loaded onto a Vivacon 500 centrifugal filtration device with MWCO 10 kDa (Sartorius Stedim Biotech). The filter was washed with 50 μL of 50 mM NaHCO_3_, and the flow-through (peptidome fraction) was collected. The peptidome fraction was split into halves. One half was reduced and alkylated by using the same amounts of the same reagents as for protein concentrate (please see below) with one additional reduction step (for iodoacetamide (IAA) inactivation); the second half was analysed without any treatment. The protein concentrate retained in the Vivacon 500 device was mixed with 100 μL of 50 mM dithiothreitol and incubated for 30 min. After additional centrifugation, 100 μL of 50 mM IAA were added and the filter was incubated in the dark for 30 min. After the next centrifugation step, the filter was washed with 200 μL of 50 mM NaHCO_3_. Trypsin (sequencing grade, Promega) was added onto the filter and the mixture was incubated for 14 h at 37 °C. The tryptic peptides were finally eluted by centrifugation followed by two additional elutions with 50 μL of 50 mM NaHCO_3_. The peptides were extracted from the vials by using 25% formic acid (FA)/acetonitrile (1:1 v/v mixture) in presence of 0.001% poly(ethylene glycol) and vacuum concentrated.

The peptide mixtures were subjected to LC–MS/MS analysis by using an RSLCnano system (Thermo Fisher Scientific, Waltham, MA, USA) coupled to an Impact II Qq-Time-Of-Flight mass spectrometer (Bruker, Bremen, Germany). Prior to LC separation, peptides were online concentrated on a trap column (100 μm × 30 mm) filled with 3.5-μm X-Bridge BEH 130 C18 sorbent (Waters, Milford, MA, USA). The peptides were separated using an Acclaim Pepmap100 C18 column (3 µm particles, 75 μm × 500 mm; Thermo Fisher Scientific) using the following solvent gradient (mobile phase A: 0.1% FA in water; mobile phase B: 0.1% FA in 80% acetonitrile; 300 nl/min): the gradient elution started at 1% mobile phase B and increased to 56% during the first 50 min, then increased linearly to 80% mobile phase B over the next 5 min, followed by isocratic elution with 80% mobile phase B for 10 min. Equilibration of the trapping column and the column was done prior to sample injection. The analytical column outlet was directly connected to the CaptiveSpray nanoBooster ion source (Bruker, Bremen, Germany). MS and MS/MS spectra were acquired using a data-dependent strategy with a 3 s long cycle time. Mass range was set to *m/z* range of 150–2200 and precursors were selected from *m/z* range of 300–2000. The acquisition speed of MS and MS/MS scans was 2 Hz and 4–16 Hz, respectively. Speed of MS/MS spectra acquisition was based on precursor intensity. The pre-processing of the mass spectrometric data including recalibration, compound detection, and charge deconvolution was carried out using DataAnalysis software (version 4.2 SR1; Bruker).

### Bioinformatic integration of proteomic and transcriptomic data

To identify venom peptides and proteins, mass spectral data were searched against the database of possible amino acid sequences obtained from the venom-gland transcriptome using the Paragon and ProtGroup algorithms in ProteinPilot software (SCIEX, Framingham, MA, USA) with a cut-off of 95% confidence at the protein level. We decided to use only the *L. murina* transcriptome in the bioinformatic data integration, but we refer to the proteome on the genus level due to possible misidentifications from unidentified *Lampona* sp. (see Discussion). cDNA sequences encoding the identified toxins were re-mapped to correct errors and identify variants and missing termini using Geneious software version 2019.0.4 (Biomatters, Auckland, New Zealand). Precursor sequences were then annotated using SignalP^66^, BLAST searches against the SwissProt database^67,68^, and HMMER searches against the Pfam database^69,70^. Because of the larger size of many of the toxins (> 5 kDa), the mature structures of very few were resolved from the native and reduced/alkylated datasets and for this reason we generated predicted mature forms for the remainder using the hidden Markov model of the SpiderP algorithm^71^. These 'best mature' sequences (Supplementary Table S1, column AE) were then used as a database for a new search of the mass spectral data using the Paragon and Protgroup algorithms, and redundant or undetected toxins were removed from the proteome. Toxins were named according to rational nomenclature guidelines^72^.

### Bioassays with crude venom

Pooled crude venoms from *Lampona* and *Gnaphosa* spiders were diluted to different concentrations in 0.1 M ammonium acetate buffer, pH 6.1 for the venom toxicity bioassays^57^. Model prey were anesthetised with CO_2_ before injection of 50 nl of venom into the thorax or the prosoma using a glass microsyringe. Several venom concentrations that caused dose/weight-dependent effects were tested; 5–30 prey were used per venom concentration (Supplementary Table S2). Simultaneously, for each concentration, a control cohort of prey were injected with ammonium acetate buffer to exclude the effect of buffer and merely piercing the prey with the capillary. If there was mortality in the control group, the data for the given trial was discarded. After injection, prey were placed individually into small Petri dishes (diameter 35 mm) with a small piece of moisturized cotton. Each tested prey individual was weighed using a Kern 770 balance (Balingen, Germany) with a precision of 0.01 mg. Mobility of the prey was observed 1, 3 and 24 h after the injection. Prey were considered dead or completely paralyzed when there was no movement after a light touch with a pincer and partially paralyzed when they could not move in Petri dish normally (not able to walk, erratic movements, etc.).

### Bioassays with venom fractions of Lampona

To investigate which venom components are responsible for the specific venom toxicity in *Lampona*, we performed bioassays with two venom fractions: smaller peptides (< 10 kDa) and larger proteins (> 10 kDa). The crude venom sample (2.25 µl) was diluted in 100 µl of 50 mM PBS and added into a Microcon 10 centrifugal filter unit (Merck Millipore, Germany) with a 10 kDa molecular weight cut-off. The low mass fraction (< 10 kDa) was obtained by centrifugation at 14,000 g. The high mass fraction (> 10 kDa), which remained on the upper part of the filter, was collected after being shaken in an additional 25 µl of 50 mM PBS.

Both fractions were diluted in ammonium acetate buffer, and one concentration for the given prey was prepared (1:50 for spider prey, corresponding venom dose: 0.56 ± 0.15 nl/mg; 1:10 for cricket prey, corresponding venom dose: 0.74 ± 0.14 nl/mg). This concentration was higher than the median lethal dose value from the previous experiment; therefore, it should induce paralysis or death in prey. Diluted venom fractions were injected into ten individuals of preferred and alternative prey types (spider and cricket) and paralysis/mortality was observed over 24 h. As a control, buffer only was injected into ten prey individuals.

### Bioassays data analysis

Venom toxicities were compared using dose–response analyses (Supplementary Fig. S1) performed in the R environment^73^. The complementary log–log model with binomial distribution using generalised linear models (GLM-b) was used to fit the binary data. The mortality of the prey after 24 h was the response variable, log-transformed venom dose (in nl per mg) was a covariate, and venom origin and prey type were factors. Median lethal dose values (LD_50_) within 24 h for each combination of the venom and prey type were estimated from models using dose.p function from the MASS package^74^. A 95% confidence interval for each LD_50_ value was calculated using the formula for normal distribution^75^.

To evaluate the paralysing properties of each venom, effective doses (ED_50_) were estimated from the models (GLM-b) where the affected prey (dead or paralysed) after 1 h was used as the response variable instead of mortality. Comparison between paralysing and lethal effect for each spider was made using another model with the type of effect (paralysis/mortality) as another factor. In the latter case, Generalised Estimating Equations with a binomial distribution (GEE-b) from the geepack package^76^ were used instead of GLM-b, as the rate of affected prey after 1 and 24 h represents repeated measurements on prey individuals. An autoregressive correlation structure (AR1) for replicated observations over time was used to account for these temporal replications^77^.

The toxicity of the *Lampona* venom fractions were also compared using a generalised linear model with a binomial distribution (GLM-b). The mortality of the prey after 24 h was the response variable, venom concentration (in nl per mg) was a covariate, and venom fraction and prey type were factors.

## Supplementary Information

Supplementary Information 1.

Supplementary Information 2.

## Data Availability

The raw reads have been submitted to GenBank, the sequence read archive (SAR) accession is SRR18933286, BioProject PRJNA832048. The Transcriptome Assembly has been deposited at DDBJ/EMBL/GenBank under the accession GJYG00000000. The mass spectrometry proteomics data have been deposited to the ProteomeXchange Consortium via the PRIDE partner repository with the dataset identifier PXD033502. Protein sequences discovered in this project were submitted to GenBank and assigned the accession numbers ON226530–ON226737. Additional data that support the findings of this study are available from the corresponding authors upon reasonable request.
