# Supplementary material for: Composition and toxicity of venom produced by araneophagous white-tailed spiders (Lamponidae: Lampona sp.﻿)
Source: Sci Rep. 2022 Dec 14;12:21597. doi: 10.1038/s41598-022-24694-5 (PMC9751281; doi:10.1038/s41598-022-24694-5)
Supplement: Supplementary file 1 — Supplementary Information 1. [file 41598_2022_24694_MOESM1_ESM.docx]

**Composition and toxicity of venom
produced by araneophagous white-tailed spiders (Lamponidae: *Lampona* sp.)**

**Supplementary material**

**Ondřej Michálek^1*^, Andrew A. Walker^2,3^, Ondrej Šedo^4, 5^, Zbyněk Zdráhal^4, 5^, Glenn F. King^2,3^ and Stano Pekár^1^**

^1^Department of Botany and Zoology, Faculty of Science, Masaryk University, Kotlářská 2, 611 37 Brno, Czech Republic

^2^Institute for Molecular Bioscience, The University of Queensland, St. Lucia, Queensland, 4072, Australia

^3^Australian Research Council Centre of Excellence for Innovations in Peptide and Protein Science, The University of Queensland, St Lucia, QLD 4072, Australia

^4^Research Group Proteomics, Mendel Centre for Plant Genomics and Proteomics, Central European Institute of Technology, Masaryk University, Kamenice 5, 625 00, Brno, Czech Republic

^5^National Centre for Biomolecular Research, Faculty of Science, Masaryk University, Kamenice 5, 625 00, Brno, Czech Republic

*Corresponding author: michalek.ondrej@mail.muni.cz

**Supplementary Table S1:** The spreadsheet containing detailed sequence, annotation, and mass spectrometric identification statistics for all identified peptides and proteins. *[Available as a separate xlsx file.]*

**Supplementary Table S2.** The number of prey (spider and cricket) injected with different concentrations of the crude venom of two spiders.

| **Concentration** | **No. of injected prey** | | | |
| --- | --- | --- | --- | --- |
|  | ***Lampona* sp.** | | ***Gnaphosa* sp.** | |
|  | **Spider** | **Cricket** | **Spider** | **Cricket** |
| 1:200 | 10 | 0 | 0 | 30 |
| 1:100 | 20 | 10 | 20 | 20 |
| 1:50 | 20 | 10 | 20 | 0 |
| 1:10 | 20 | 30 | 20 | 20 |
| pure venom | 0 | 5 | 0 | 0 |
| **TOTAL** | **70** | **55** | **60** | **70** |


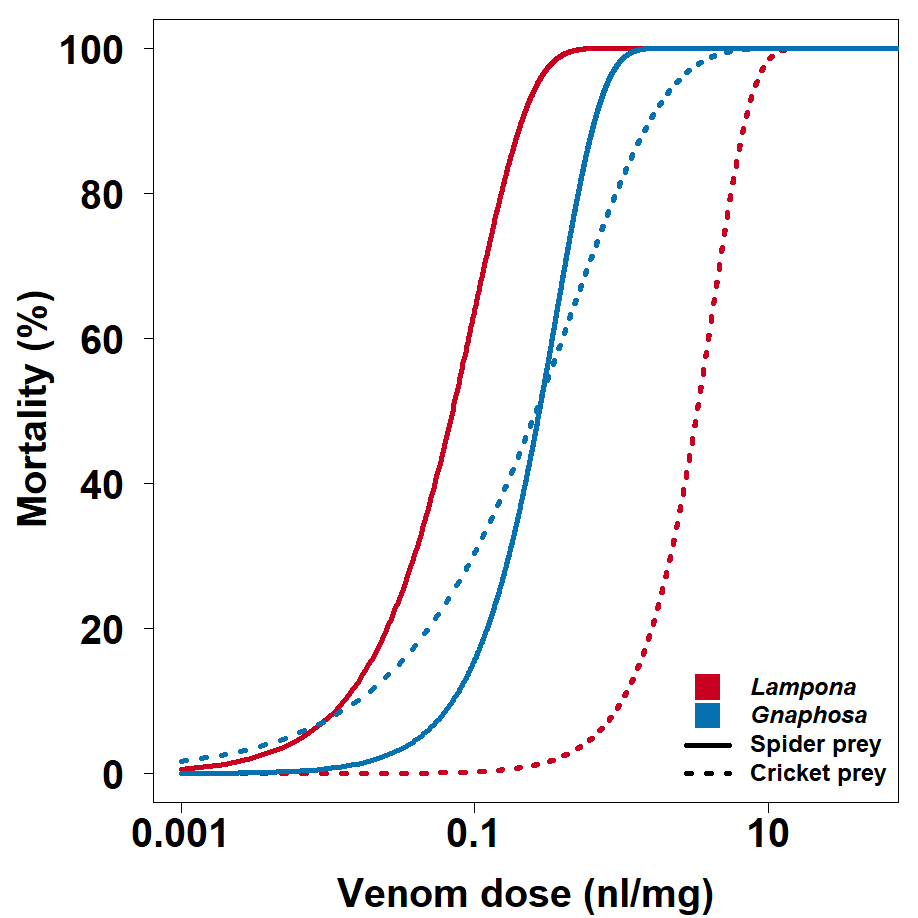


**Supplementary Figure S1.** Dose-response estimated models showing the relationship between prey mortality (measured 24 h after venom injection) and the range of crude venom doses of *Lampona* sp. and *Gnaphosa* sp..


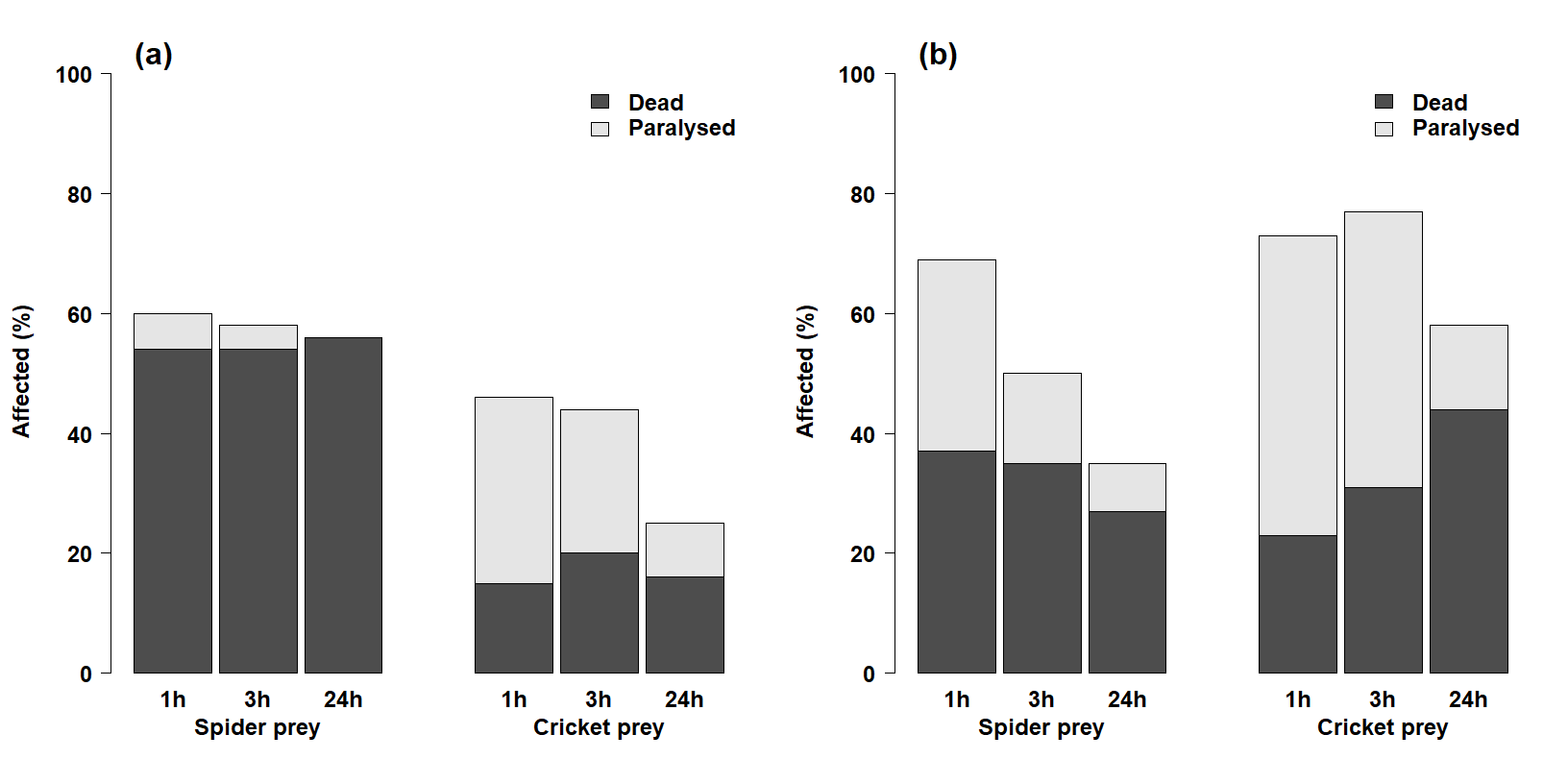


**Supplementary Figure S2.** Comparison of the proportion of affected (dead or paralysed) prey individuals injected with various venom concentrations of *Lampona* sp. (**A**) and *Gnaphosa* sp. (**B**) crude venoms (**Supplementary Table S2**), measured at 1, 3 and 24 h.
